# Effect of Vitamin D_3_ on Lung Damage Induced by Cigarette Smoke in Mice

**DOI:** 10.1515/med-2019-0096

**Published:** 2019-11-07

**Authors:** Xin Zheng, Nini Qu, Lina Wang, Guoli Wang, Rui Jiao, Hu Deng, Sijia Li, Yibing Qin

**Affiliations:** 1 Respiratory Department, Affiliated Hospital of Liaoning University of Traditional Chinese Medicine, NO.33 Beiling Street, Huanggu District, Shenyang, 110032, Liaoning, P.R.China; 2 Liaoning University of Traditional Chinese Medicine, Shenyang, 110847, Liaoning, P.R.China

**Keywords:** Vitamin D3, cigarette smoking, lung damage, mice, over-expression

## Abstract

Cigarette smoking is known to induce serious lung diseases, but there is not an effective method to solve this problem. The present study investigated vitamin D3 on over-expression of CXCR3 and CXCL10 in mice induced by cigarette smoking. A pulmonary airway model was designed, and morphological assessment of emphysema, IL-4, IFN-γ and CXCL10 concentration in bronchoalveolar lavage fluids, expression of CXCR3 and CXCL10 were detected. Emphysema of the mice only exposed to cigarette smoke was significant, and concentration of IL-4, IFN-γ and CXCL10 was also increased. In addition, CXCR3 and CXCL10 were over-expressed. The degree of emphysema, concentration of IL-4, IFN-γ and CXCL10, and expression of CXCR3 and CXCL10 in mice administrated with low dose vitamin D3 were similar to the normally treated mice. Low dose of vitamin D3 can effectively protect the lung from the damage induced by cigarette smoke.

## 1 Introduction

Cigarette smoking is a worldwide behavior, and it is popular in all countries, across all age groups, and especially in the male population. Despite well known devastating health impact of cigarette smoke and ongoing efforts to reduce smoking prevalence, approximately 1.25 billion people smoke cigarette daily worldwide, representing one-sixth of the world’s population [1,2]. Cigarette smoking is responsible for 90% of all lung cancers, and also the major cause of chronic obstructive pulmonary disease (COPD), which will become the third leading cause of death by 2020 [3, 4, 5]. Furthermore, about half of all smokers will develop COPD or cardiovascular disease, and about 1-5% of smokers will develop a smoking-related malignancy, mostly lung adenocarcinoma or other epithelial cell tumors. Cigarette smoke exposure is the main cause of lung inflammation, which disrupts normal repair and defense mechanisms (resulting in small airway fibrosis) leading to progressive airflow limitation and induces parenchymal tissue destruction (resulting in emphysema) [6].

CXCR3 is a seven trans-membrane-spanning G protein-coupled receptor, which is mainly expressed on activated T and B lymphocytes, dendritic and macrophage cells, and natural killer (NK). Previous papers have reported that CXCR3 and its interferon (IFN)-inducible CXC chemokine ligands CXCL10 (IFN-gamma-inducible 10000 molecular weight protein/CXCL10) played an important role in regulating leukocyte trafficking to the lung inflammation [7, 8, 9, 10]. Inflammation is associated with secretion of CXCL10 from leukocytes, eosinophils, neutrophils, monocytes, epithelia, endothelial and stromal cells, and keratinocytes in response to IFN-γ. CXCL10 induces chemotaxis, apoptosis, cell growth inhibition and angiostasis, and CXCL10 concentrations are significantly up-regulated in patients with asthma, COPD, pulmonary tuberculosis, and other airway inflammatory diseases. Furthermore, the CXCL10-CXCR3 axis represents a potential pharmacologic target for various human diseases, including cancer and infectious diseases [11,12].

Currently, some papers have reported that vitamin D may have anti-cancer ability in a wide range of cancers as well as against metastasis and progression [13]. Moreover, in various tumor models, vitamin D has the ability to act against metastasis, including the lung, melanocyte, retina, bone, colon, kidney, breast and prostate cancers. Under ultraviolet radiation, vitamin D can be hydroxylated in the liver to produce 25(OH)D, which can then be converted into 1,25(OH)_2_D (D_3_) by 1-α-hydroxylase. A number of studies have demonstrated that vitamin D_3_ is more active than others, and plays a possible protective role in the progression of breast, colon and prostate cancer [14,15]. Furthermore, a high level of vitamin D_3_ might reduce the risk of cancer death. The mechanism may be explained that many cell types contain vitamin D receptors in organs of human body, when these receptors are activated by vitamin D_3_, they induce differentiation and inhibit invasiveness, metastatic, proliferation and angiogenesis potential.

Previous studies have investigated cigarette smoke induced pulmonary inflammation in mice, and chronic cigarette smoking caused hypertension, increased oxidative stress, an impaired NO bioavailability, and endothelial dysfunction. However, there is no any kind of bioactive material that has been found with the ability to inhibit the lung diseases induced by cigarette smoke exposure. Therefore, the aim of our work was to study an influence of vitamin D_3_ on over-expression of CXCR3 and CXCL10 in mice induced by cigarette smoke, and understand whether vitamin D_3_ has the ability to protect the lung under long time cigarette smoke exposure.

## 2 Materials and methods

### 2.1 Mice

80 of Male BALB/c mice (sterile grade, weight 21.2 ± 2.3 g，age 2.1±1.3s) were purchased from Experimental Animal Center, Shanghai Jiao Tong University School of Medicine, and kept in a temperature and humidity controlled room (50%, 25 °C) with free access to standard water and food. There was no statistically significant difference of vitamin D levels in all mice.

### 2.2 Experimental and pulmonary airway model design

After 7 days of adaptation, the mice were randomly divided into 5 groups consisting of 16 animals each: 0group: Control group: mice administered with water and food without cigarette smoke exposure and vitamin D_3_ administration. 1group： Cigarette smoke exposure group: mice administered with water and food, and cigarette smoke exposure without vitamin D_3_ administration. 2group：vitamin D_3_ treatment group one: mice administered with water and food, and cigarette smoke exposure and then vitamin D_3_ drops at a concentration of 50mg/kg were given by the stomach (1000u). 3group: vitamin D_3_ treatment group two: mice administered with water and food, and cigarette smoke exposure and then vitamin D_3_ drops at a concentration of 50mg/kg were given by the stomach (2000u). 4group: vitamin D_3_ treatment group three: mice administered with water and food, and cigarette smoke exposure and then vitamin D_3_ drops at a concentration of 50mg/kg were given by the stomach (3000u).

**Figure 1:**
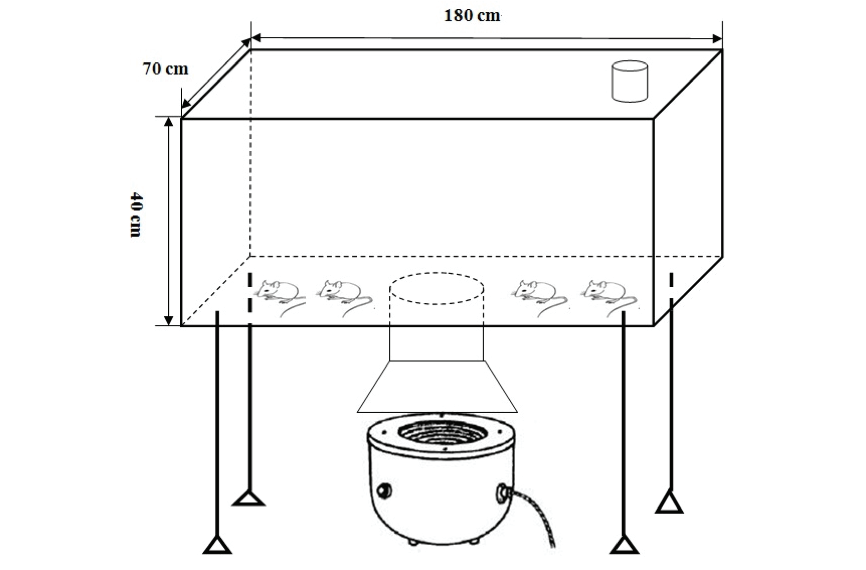
The designed pulmonary airway model.

These mice were exposed for 60 min (30 min in a.m., 30 min in p.m.) per day at 6 consecutive days per week (16 weeks) in a pulmonary airway model (Fig. 1). All animal procedures were in accordance with the guidelines for care and use of laboratory animals and were approved by the Animal Experimentation Ethics Committee of Shanghai Jiao Tong University (Shanghai, China).

### 2.3 Morphological analysis

On the last day of the 16^th^ week after exposure to cigarette smoke, mice in each group were sacrificed, and the lungs were removed for morphological and morphometric assessment of emphysema.

### 2.4 Determination of IL-4, IFN-γ and CXCL10 in bronchoalveolar lavage fluids (BALF)

Concentrations of IL-4 and IFN-γ in bronchoalveolar lavage fluids (BALF) were measured using mouse ELISA kits (the ABC-ELISA method) obtained from Westang Biotechnology (Shanghai, China), according to the directions. Absorbance of the final solution was determined at 450 nm using a microplate reader (Plus384, MD Spectra-Max, USA).

### 2.5 Expression of CXCR3 and CXCL10 in lungs

Total RNA was extracted from the lung cells by using TRIzol miRNA isolation reagent (Invitrogen, Carlsbad, USA). The concentrations of extracted RNA were determined by a NanoDrop ND-1000 Spectrophotometer (Saveen Werner, Malmö, Sweden) and stored at -80 °C until use. Reverse transcription was performed using the PrimeScript^TM^ RT regent Kit (TaKaRa Biotechnology, China) according to the manufacture’s protocol. Primers for CXCR3, CXCL10 and β-actin were as follows, CXCR3

forward primer: 5’-CCGTCCAGTGGGTCTTTGG-3’, reverse primer: 5’-CTGCGTAGAAGTTGATGTTGAAGAG-3’. CXCL10 forward primer: 5’-GCTGCCGTCATTTTCTGC-3’, reverse primer: 5’-TCTCACTGGCCCGTCATC -3’. β-actin forward primer: 5’-TGTCCACCTTCCAGCAGATGT-3’, reverse primer: 5’-AGCTCAGTAACAGTCCGCCTAG-3’. PCR condition was 50 °C, 2 min; 95 °C, 10 min; 40 cycles of 95 °C, 15 s; 60 °C, 60 s. CXCR3 and CXCL10 expression was normalized to the housekeeping gene β-actin and calculated relative to the control. The calculation formula was as follow:

ΔCt = Ct (gene)－Ct (β-actin)

Expression value = 2^－ΔCt^

### 2.6 Statistical analysis

Toxicological experiments were conducted at least in triplicate. Data were analyzed by analysis of variance and using SPSS software (SPSS for Windows, 16.0, 2007, SPSS Inc., USA), and reported as mean ± standard deviation (SD). Significant differences were determined using Duncan’s multiple-range test at *P*<0.05.

## 3 Results

### 3.1 Morphological assessment of emphysema

**Figure 2:**
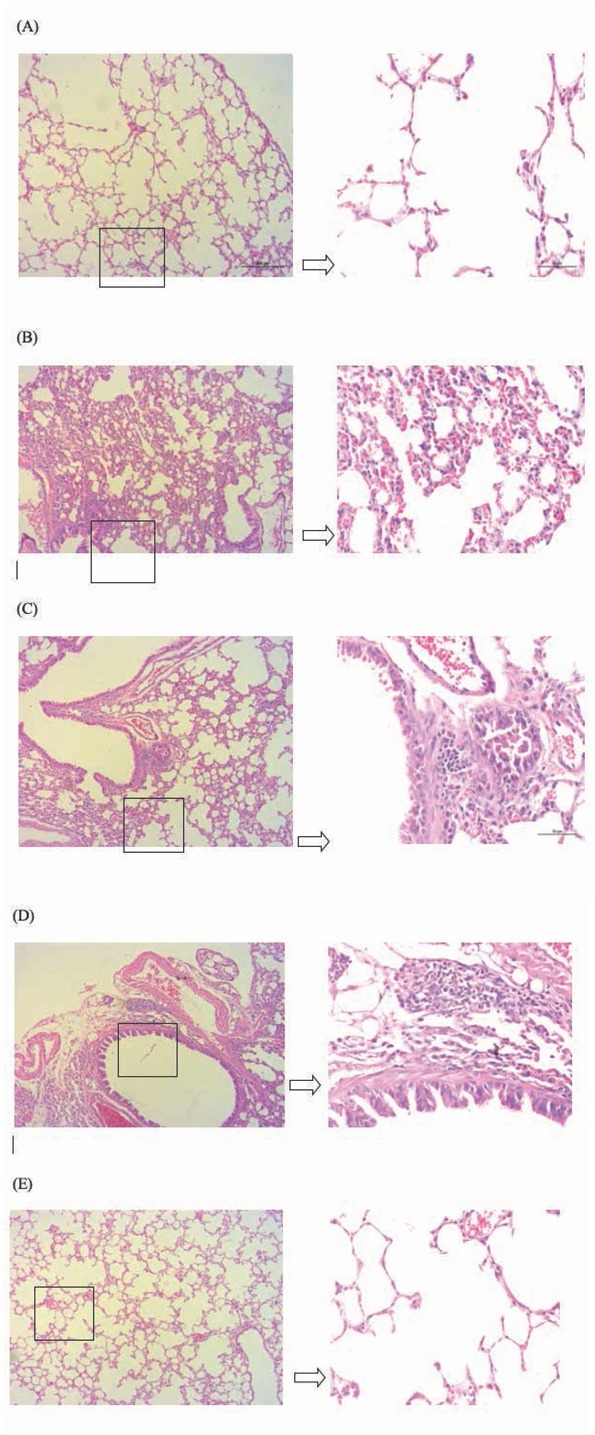
Morphological assessment of emphysema. (A) The lung of the mice only exposed to cigarette smoke; (B) the lung of the mice exposed to normal air (control); (C) the lung of the mice exposed to cigarette smoke and administrated with 1000u of vitamin D_3_; (D) the lung of the mice exposed to cigarette smoke and administrated with 2000u of vitamin D_3_; (E) the lung of the mice exposed to cigarette smoke and administrated with 3000u of vitamin D_3_.

The degree of emphysema as assessed by morphology in the mice with different treatment is shown in Fig. 2. The mice only exposed to cigarette smoke showed a significant degree of emphysema (ampliative alveolar space), and developed an obvious lung vascular remodeling (alveolar break and blend into big vesicle) and pulmonary hypertension (decreased alveolar amount) Fig. 2A. The degree of emphysema, lung vascular remodeling and pulmonary hypertension of the mice administrated with 2000u of vitamin D_3_ after cigarette smoke exposure was similar to the mice administrated with 3000u of vitamin D_3_ (Fig. 2D,E). The lung of the mice in the control group and the mice administrated with 1000u of vitamin D_3_ after cigarette smoke exposure showed a well-fixed normal parenchyma (Fig. 2B,C).

### 3.2 Determination of IL-4, IFN-γ and CXCL10 in BALF

**Figure 3:**
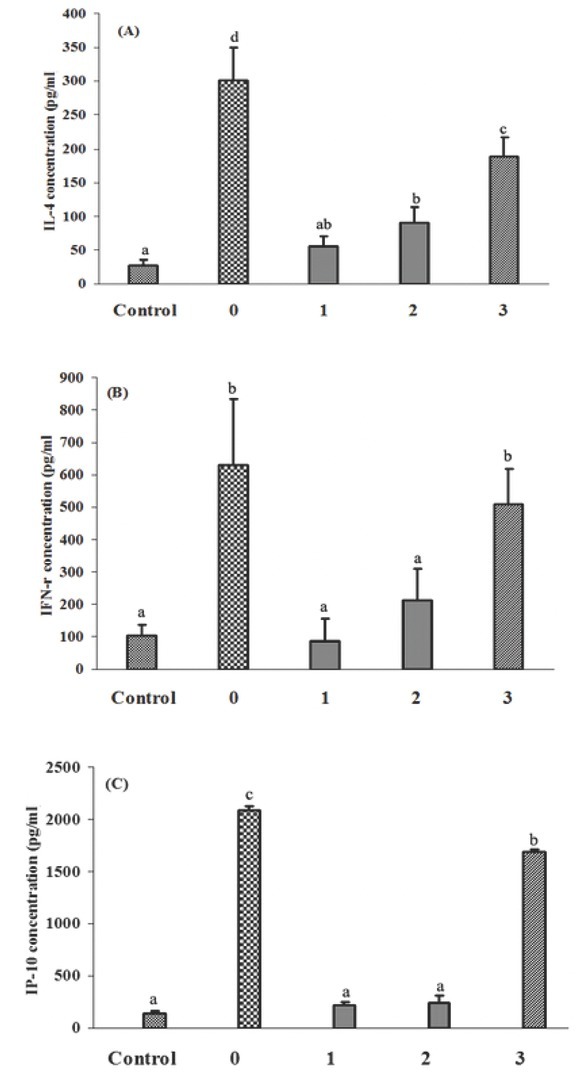
The concentrations of IL-4, IFN-γ and CXCL10 in BALF. (A) The concentration of IL-4 in BALF; (B) The concentration of IFN-γ in BALF; (C) The concentration of CXCL10 in BALF. Different letters denoted significantly different (*P*< 0.05).

The concentrations of IL-4, IFN-γ and CXCL10 in BALF of differently treated mice are shown in Fig. 3 (A, B and C).

IL-4 concentration in the 0 group was significantly higher than that in the other groups (*P*<0.05), and IL-4 concentration in the 3 group was significantly different from other (*P*<0.05). No significant difference was found between the 1, 2 groups and the control group (*P*>0.05). The difference of IFN-γ concentration in each group was similar to the IL-4 concentration, and no significant difference was discovered between the 1, 2 and control groups (*P*>0.05), and there was no significant difference between the 0 and 3 groups (*P*>0.05). Furthermore, the difference of CXCL10 concentration in each group was absolutely similar to the

IFN-γ concentration. The concentrations of IL-4, IFN-γ and CXCL10 in BALF of the mice administrated with 1000u or 2000u of vitamin D_3_ after cigarette smoke exposure was significantly lower than that in the mice only exposed to cigarette smoke.

### 3.3 Expression of CXCR3 and CXCL10 in lungs

**Figure 4:**
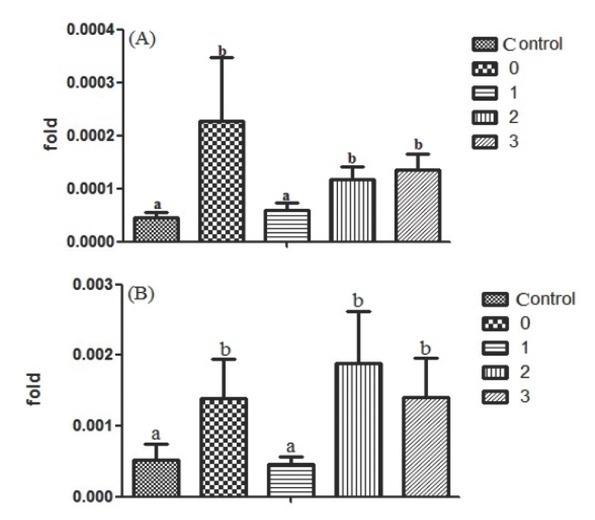
The gene expression of CXCR3 and CXCL10 in the lung tissue. (A) The expression of CXCR3 in lung of the mice with different treatments; (B) The expression of CXCL10 in lung of the mice with different treatments. Different letters indicated significantly different (P< 0.05).

Fig. 4A and Fig. 4B show the expression of CXCR3 and CXCL10 in lungs of the differently treated mice. The expression of CXCR3 in the 0 group was significantly higher than that in the control and 1 groups (*P*<0.05). No significant difference was found between the 0, 2 and 3 groups (*P*>0.05). The representation of CXCL10 expression in each group was entirely similar to CXCR3. The expression of CXCR3 and CXCL10 in lung of the mice administrated with 1000u of vitamin D_3_ after cigarette smoke exposure was significantly lower than that in the mice only exposed to cigarette smoke. No significant difference was observed between the mice administrated with 2000u or 3000u of vitamin D_3_ and the mice only exposed to cigarette smoke.

## 4 Discussion

Herein, we report that the mice exposed to cigarette smoke can develop serious emphysema. The results were similar to pervious study, in which pulmonary emphysema in mice exposed to cigarette smoke was also observed [16]. According to pervious study, emphysema may be caused by the accumulation of lipid peroxidation and matrix degradation products, and presence of carbon monoxide (CO) in cigarette smoke [17]. Furthermore, the administration of low dose of vitamin D_3_ had the ability to inhibit the degree of emphysema. However, the administration of higher dose of vitamin D_3_ did not have better effect as we expect, in contrast the degree of emphysema was also significant. It is well known that the active vitamin D_3_ has the ability to modulate lymphocyte and macrophage functions, and prevent monocyte differentiation and maturation [18]. The degree of emphysema in the mice administrated with vitamin D_3_, therefore, was not so significant. However, no negative correlation has been found between the concentrations of vitamin D_3_ and the degree of emphysema in mice.

CXCR3 belongs to the CXC chemokine receptor subfamily and has three endogenous ligands such as CXCL9, CXCL10, and CXCL11. CXCR3 has been detected in many malignant tumors and is associated with patient outcomes. The CXCR3 expression was positively correlated with tumor thickness and the presence of distant metastases [19,20]. Moreover, CXCL10 is also involved in some biological processes and plays an essential role during the inflammatory response. CXCL10 had been originally identified as a chemokine that modulated innate and adaptive immune responses and mediated leukocyte trafficking. Moreover, the alteration of CXCL10 has been related to pathogenesis during chronic inflammation, infectious and autoimmune diseases [21, 22, 23]. The results demonstrated that CXCR3 and its ligand (CXCL10) had the ability to mediate lung damage in mice induced by cigarette smoke. Hence, the over- expression of CXCR3 and CXCL10 can be detected in the lung of mice only exposed to cigarette smoke. However, the active vitamin D_3_ also has the ability to prevent, or markedly suppress, autoimmune diseases and inflammatory activity. Therefore, the expression of CXCR3 and CXCL10 may not be so high, and the low dose vitamin D_3_ can keep the expression at a normal level.

The mRNA levels of CXCL10 and CXCR3 were strongly correlated, and they also correlate very well with other established markers of inflammation, including IL-4 and IFN-γ [24]. Hence, the over-expression of CXCL10 and CXCR3 would induce the concentrations of IL-4 and IFN-γ increased at the same time.

In conclusion, cigarette smoking can activate the immune response to protect our body, and induce the over-expression of related mRNA and some chemokine, including CXCL10, CXCR3, IL-4 and IFN-γ. At the same time, the immune system is damaged gradually. The administration of vitamin D_3_ can cooperate with immune system to defend the damage. Moreover, our data indicate that vitamin D_3_ may modulate the immune system, and protect the lung from damage induced by cigarette smoke.
